# Small Bowel Perforation Secondary to Portal Vein Thrombosis

**DOI:** 10.7759/cureus.25911

**Published:** 2022-06-13

**Authors:** Arthur Cecchini, Ahmad Othman, Koushik Sanku, Amanda Cecchini, Deidra Pierce

**Affiliations:** 1 Internal Medicine, East Tennessee State University Quillen College of Medicine, Johnson City, USA

**Keywords:** surgery general, adult gastroenterology, intraabdominal abscess, super morbid obesity, small bowel resection, superior mesenteric vein thrombosis, portal venous thrombosis

## Abstract

Portal vein thrombosis (PVT) is a heterogeneous entity often described as either an acute or chronic occlusion of the portal vein or its tributaries. The clinical presentation is highly variable, and it often mimics other more common causes of abdominal pain. In most patients, imaging studies such as doppler ultrasound, computed tomography, or magnetic resonance imaging are adequate for diagnosis. Occasionally imaging studies may be inadequate, and the diagnosis may not be made until complications such as bowel necrosis and perforation have occurred. We present a case of a morbidly obese 45-year-old female who was initially treated for suspected small bowel enteritis and discharged home on several occasions after nonspecific findings on abdominal imaging were seen and interval improvement in symptoms occurred with intravenous fluids and antibiotics. She then presented with worsening symptoms and was found on abdominal imaging to have a large fluid collection in the peritoneal cavity requiring exploratory laparotomy with peritoneal washout and partial small bowel resection due to perforation. She was diagnosed with PVT with mesenteric extension after samples of the resected mesentery were evaluated in the pathology laboratory. Her treatment included a prolonged course of antibiotics, total parenteral nutrition, and anticoagulation.

## Introduction

Portal vein thrombosis (PVT) is defined as either an acute or chronic thrombotic occlusion of the portal vein or its tributaries. This disease is most often seen in patients with cirrhosis, hepatic carcinoma, or thrombophilia [1,2]. The clinical presentation may be highly variable and depends on the size, chronicity, and if an extension of the thrombus into the mesenteric vessels is present. In acute PVT, patients may present with a rapid accumulation of ascites and vague abdominal discomfort. If ischemia, necrosis, or perforation is present, patients may present acutely ill with fever, abdominal pain, and hemodynamic instability. Chronic thrombosis often presents insidiously and may not be detected until complications such as variceal bleeding or extension into the mesenteric vessels occur [2-5]. Treatment is variable and depends on whether cirrhosis is present, if the occlusion is acute or chronic, if extension into the mesentery is present, or if the occlusion of the portal vein is partial or complete. When indicated, therapy generally includes anticoagulation for at least six months or indefinitely in those with thrombophilia [4-6]. In morbidly obese patients, this disease may be exceedingly difficult to diagnose with imaging studies alone. Obstacles to obtaining a diagnosis include poor image quality as well as patients exceeding the size and weight limits for magnetic resonance imaging (MRI) or computed tomography (CT) machines. When adequate imaging is unable to be obtained, PVT may not be diagnosed until complications have already arisen and specimens of the mesentery are obtained during the surgical intervention [3]. Prompt diagnosis is imperative, as bowel ischemia, necrosis, and perforation may occur without timely anticoagulation if the thrombus extends into the superior mesenteric vein and its tributaries [2].

## Case presentation

A 45-year-old female with a past medical history of hereditary spherocytosis status post remote splenectomy, gastroesophageal reflux disease, obstructive sleep apnea, nonalcoholic steatohepatitis, hypertension, previous cholecystectomy, and morbid obesity presented to the hospital with complaints of severe abdominal pain, nausea, vomiting, and constipation progressively worsening for eight days.

**Figure 1:**
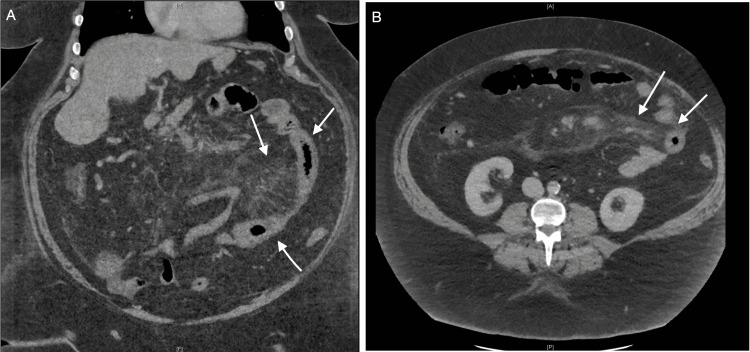
CT showing thickened loops of small bowel suggestive of enteritis and fatty stranding around the mesenteric vessels CT: computed tomography (A) Coronal view. (B) Axial view

She was seen in the emergency department (ED) three times over the past 20 days and was admitted after the third emergency department visit for similar, yet less severe symptoms. A CT scan of the abdomen and pelvis with intravenous contrast was performed at two of the three ED visits. The first showed thickening of the small bowel in the left abdomen suggestive of enteritis, fatty stranding around central mesenteric vessels likely related to vascular congestion (chronic finding seen on remote previous CT), and patchy fatty stranding throughout the abdomen. The portal vein was not mentioned in the CT report (Figures 1A, 1B).

**Figure 2:**
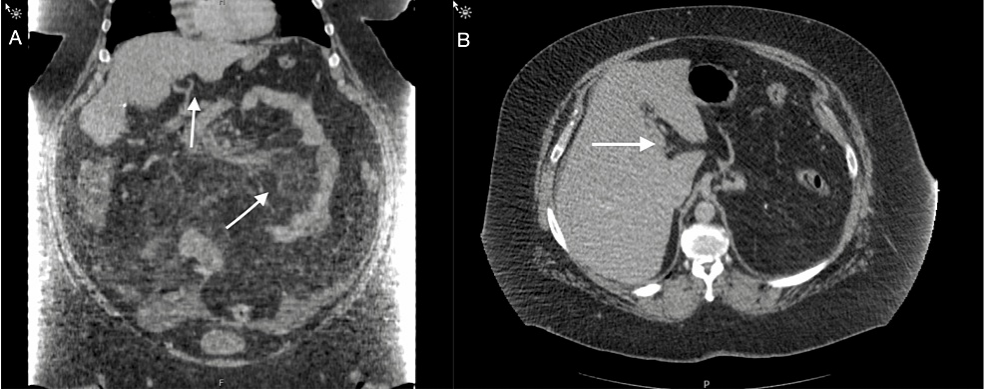
CT showing hazy mesentery, an unremarkable small bowel, and nonvisualization of the portal vein CT: computed tomography (A) Coronal view. (B) Axial view

Repeat CT of the abdomen and pelvis with intravenous contrast three days later showed nonvisualization of the main portal vein and haziness of the central mesentery consistent with venous congestion of the superior mesenteric vein. The small bowel itself was unremarkable (Figures 2A, 2B).

**Figure 3:**
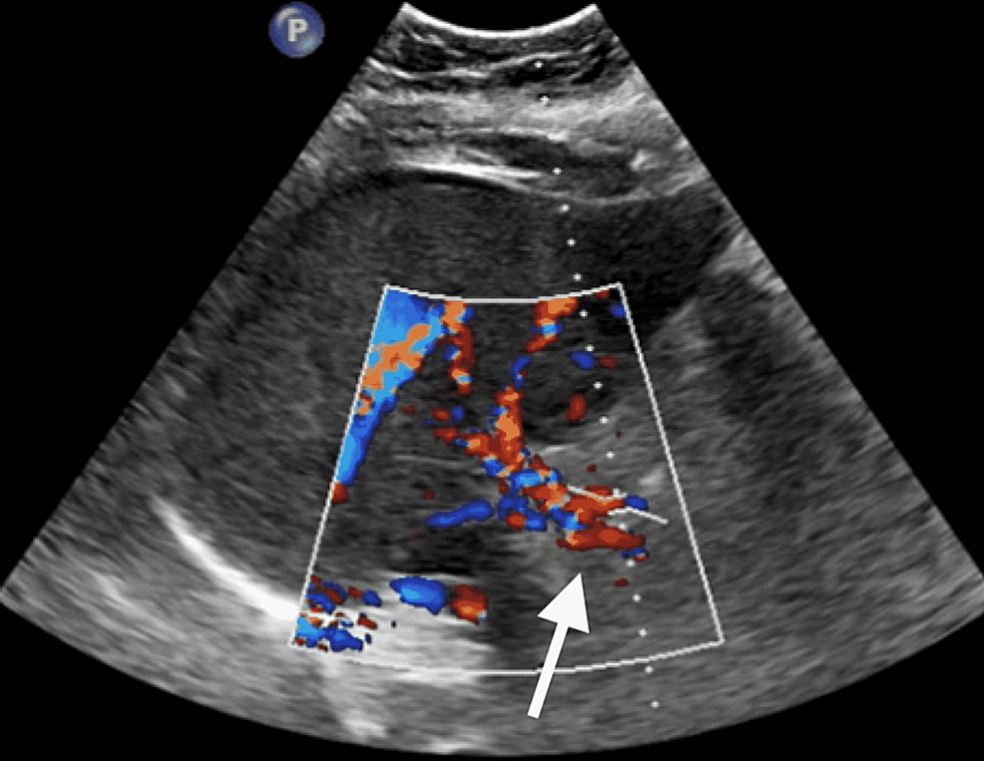
Liver US showing grossly patent main portal vein flow US: ultrasound

An ultrasound of the liver with duplex imaging was also performed showing grossly present main portal vein flow but was unable to confirm or exclude PVT (Figure 3).

During each of these hospital visits, the patient had symptomatic improvement with intravenous fluids, antibiotics, and antiemetic therapy. Upon discharge, she was tolerating oral intake and eager to go home.

**Figure 4:**
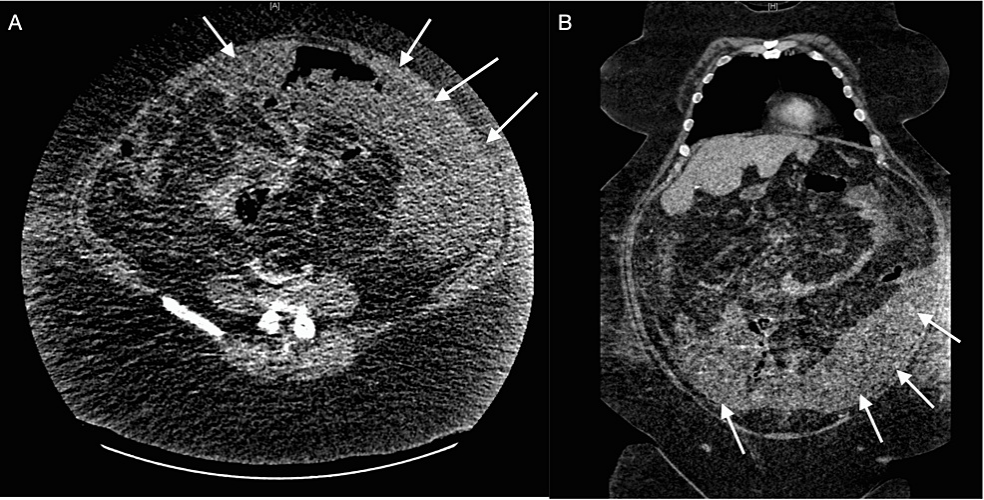
CT of the abdomen and pelvis showing 32x12x23 cm complex loculated fluid collection with scattered air pockets CT: computed tomography (A) Axial view. (B) Coronal view

When she presented a fourth time with similar but much more severe symptoms, she had a third CT of the abdomen and pelvis performed and was found to have a 32x12x23 cm complex loculated fluid collection with scattered air pockets compatible with a large intraabdominal abscess/fluid collection (Figures 4A, 4B).

Vital signs showed a temperature of 98.7° F, blood pressure 108/77 mmHg, heart rate of 122 per minute, respiratory rate of 19 per minute, and body mass index of 70 kg/m^2^. Laboratory studies were significant for low potassium, sodium, and chloride, elevated total bilirubin, normal serum lactate, high leukocyte count with an increase in neutrophils, and high platelet count (Table 1).

**Table 1:** Laboratory results on presentation

Laboratory studies	Patient values	Reference values
Potassium (mEq/L)	2.8	3.5-5.0
Sodium (mEq/L)	135	136-145
Chloride (mEq/L)	97	98-106
Total bilirubin (mg/dL)	1.1	0.3-1.0
Lactate, serum (mmol/L)	1.4	0.7-2.1
Leukocyte count (μL)	19,800	4,000-11,000
Platelet count (μL)	834,000	150,000-400,000

Electrolyte replacement was administered and piperacillin-tazobactam was initiated. She was taken to the operating room for emergent intraabdominal washout of greater than four liters of intestinal contents, resection of a segment of the proximal to mid-ileum due to perforation and subsequent enteroenterostomy anastomosis. A negative pressure dressing was placed after surgery. Infectious disease was consulted, and micafungin was added for antifungal coverage.

Multiple specimens from the perforation site and mesentery were taken during the patient’s laparotomy. The specimens showed venous congestion and were negative for malignancy. Sections of the mesentery were sampled, and the patient was found to have a complex thrombosis in the tributaries of the superior mesenteric vein. She was started on full-dose enoxaparin which was then transitioned to apixaban.

The hospital course was complicated by ampicillin-sensitive Enterococcal bacteremia likely secondary to intraabdominal infection/fluid collection for which the patient was continued on piperacillin-tazobactam. The patient also required follow-up surgical exploration and intraabdominal drain placement due to an anastomotic leak for which intraperitoneal drains were placed. Piperacillin-tazobactam was changed to meropenem later in the hospital course by infectious disease to remove the sodium load of piperacillin-tazobactam as the patient had developed diffuse anasarca, and the micafungin was discontinued. She required total parental nutrition (TPN) to allow time for her anastomosis to heal and was ultimately discharged with home health, TPN, and close surgical follow-up.

## Discussion

PVT is defined as either a partial or complete thrombotic occlusion of the portal vein. PVT is often described as either acute (occurring within the previous six months) or chronic (obstruction persisting greater than six months). The timeframe from onset to diagnosis is often difficult to delineate, thus this classification scheme is somewhat arbitrary. Other classifications for PVT include completely occlusive, partially occlusive, minimally occlusive, and with or without cavernous transformation [1].

The incidence and prevalence of PVT are difficult to determine. In general, patients with cirrhosis have a much higher prevalence than those without cirrhosis with studies citing prevalence rates between 1.3% and 9.8% in patients with cirrhosis [1]. One Japanese study cites a 0.05% prevalence in patients without cirrhosis [7]. Another study in Sweden cites a total prevalence of about 1%, with most cases attributable to cirrhosis or hepatic carcinoma [2,8].

Risk factors for PVT include cirrhosis, hepatic carcinoma, tumors obstructing the portal vein, Budd-Chiari syndrome, Factor V Leiden mutation, prothrombin gene mutation, Protein C deficiency, Protein S deficiency, Antithrombin III deficiency, myeloproliferative disorders, antiphospholipid syndrome, and hyperhomocysteinemia [1,2].

The clinical presentation of PVT is highly variable and often depends on the acuity and size of the thrombosis. Patients with acute PVT may present with a rapid accumulation of ascites or an acute abdomen concerning intestinal ischemia or bowel perforation. Fever and signs of peritonitis may suggest intestinal ischemia or infarction [3]. Nonspecific symptoms such as abdominal discomfort, anorexia, nausea, and early satiety may also be the presenting syndrome of acute PVT in patients without bowel ischemia or infarction. Some patients remain asymptomatic leading to a delay in diagnosis [2,4,5].

In chronic PVT, patients are often asymptomatic while the progression of portal hypertension develops. Patients may not be diagnosed until they suffer from a variceal bleed, obstructive jaundice, cholangitis, or choledocholithiasis. [2,4].

Since the obstruction is prehepatic and the hepatic artery can partially compensate for the decrease or absence of portal venous flow, liver function studies are often normal in patients with PVT, excluding patients with cirrhosis or hepatic carcinoma. Slight elevations in alkaline phosphatase or gamma-glutamyl transpeptidase may be present if concomitant portal biliopathy is present. A mildly prolonged international normalized ratio may also be present [2].

Diagnosis of PVT may be achieved with an ultrasound of the liver which has a sensitivity and specificity ranging from 80% to 100% [5]. Hyperechoic material may be detected in the portal vein, though an acute PVT may also be anechoic, thus the use of Doppler ultrasound is often required. The sensitivity and specificity of color doppler have been estimated to be 89% and 92%, respectively [2]. Other imaging modalities include CT angiography (CTA) and magnetic resonance angiography (MRA) [2,5]. The accuracy of CTA is estimated to range from 90% to 99% while the sensitivity and specificity of MRA have not yet been determined [6,9]. CTA or MRA may be useful in determining the clot burden and if extension into the mesenteric vessels is present. Determination of bowel infarction, perforation, or other complications is also better demonstrated on CT and MR imaging [2,3,5]. Occasionally PVT with extension into the mesenteric vessels may not be diagnosed until a laparotomy is performed after complications such as bowel perforation have already occurred [3].

Treatment of PVT is aimed toward the resolution of the thrombus, prevention of extension into the mesenteric vessels, and treatment of complications that may have arisen secondary to PVT [1]. Treatment strategies vary but typically include anticoagulation for six months in patients without a demonstrated thrombophilia and indefinitely in patients with thrombophilia [1,4,6].

The American College of Gastroenterology Clinical Guidelines recommends differing treatments based on whether cirrhosis is present. In patients with cirrhosis and an acute complete main PVT, isolated mesenteric vein thrombosis, or PVT with extension into the mesenteric vasculature, anticoagulation is recommended. Chronic PVT in patients with cirrhosis is treated only if there is evidence of inherited thrombophilia, progression of thrombus, or a history of bowel ischemia due to thrombus extension into the mesenteric vessels. Patients awaiting a liver transplant may also be considered for anticoagulation therapy. In general, anticoagulation is not recommended for patients with PVT related to hepatic carcinoma as it is not beneficial. It is also reasonable to perform an esophagogastroduodenoscopy in patients with cirrhosis and a chronic PVT to determine the risk of variceal bleeding before the initiation of anticoagulation. Nonselective beta-blockers should be considered for all patients with high-risk varices without a contraindication to beta-blocker therapy. Select patients may also require variceal banding before the initiation of anticoagulation [6].

In patients without cirrhosis, anticoagulation is recommended for all patients with an acute symptomatic PVT without contraindications to this treatment. Chronic PVT in patients without cirrhosis should be treated if there is suspicion or documented evidence of an inherited or acquired thrombophilia, an extension of the thrombus into the mesenteric vessels, or if current or previous bowel ischemia has occurred due to the PVT. Similar to that patients with cirrhosis, patients without cirrhosis and chronic PVT being considered for anticoagulation should be considered for esophagogastroduodenoscopy for quantification of esophageal bleeding risk. Nonselective beta-blockers and/or intervention should be considered for high-risk varices [6].

Guidelines vary regarding which method of anticoagulation should be used. The American College of Gastroenterology recommends low-molecular-weight heparin (LMWH) for most patients with or without cirrhosis for initial therapy. Warfarin or continuation of LMWH may be used for long-term therapy. In patients with significant renal insufficiency, unfractionated heparin may be used in place of LMWH for initial treatment. The American Association for the Study of Liver Diseases mentions direct-acting oral anticoagulants as an emerging option and recommends expert consultation when considering this class of anticoagulation [1,3,6].

Other options for treatment include thrombolytic therapy and thrombectomy, though further studies are needed to determine if these treatment methods are superior to early anticoagulation alone. Typically, these procedures are reserved for acute PVT and are more often used when anticoagulation alone fails to prevent the extension of the thrombus [1-3,10].

## Conclusions

PVT should be considered in patients presenting with recurrent abdominal pain and nonspecific imaging findings such as nonvisualization of the portal vein, mesenteric haziness, and bowel wall enhancement. The signs and symptoms of PVT are highly variable, and patients often present with recurrent nonspecific abdominal complaints. Noninvasive imaging studies may not be adequate in morbidly obese patients, and occasionally the final diagnosis is not able to be made until tissue specimens obtained during laparotomy are evaluated by a pathologist. The decision to provide anticoagulation without a definitive diagnosis is difficult and should be considered on a case-to-case basis to prevent the complications seen in this patient such as bowel ischemia, necrosis, and perforation.
